# *pncA* Gene Mutations Associated with Pyrazinamide Resistance in Drug-Resistant Tuberculosis, South Africa and Georgia 

**DOI:** 10.3201/eid2303.161034

**Published:** 2017-03

**Authors:** Salim Allana, Elena Shashkina, Barun Mathema, Nino Bablishvili, Nestani Tukvadze, N. Sarita Shah, Russell R. Kempker, Henry M. Blumberg, Pravi Moodley, Koleka Mlisana, James C.M. Brust, Neel R. Gandhi

**Affiliations:** Emory University Rollins School of Public Health and School of Medicine, Atlanta, Georgia, USA (S. Allana, R.R. Kempker, H.M. Blumberg, N.R. Gandhi);; Rutgers University—Public Health Research Institute, Newark, New Jersey, USA (E. Shashkina);; Columbia University Mailman School of Public Health, New York, New York, USA (B. Mathema);; National Center for Tuberculosis and Lung Diseases, Tbilisi, Georgia (N. Bablishvili, N. Tukvadze);; Centers for Disease Control and Prevention, Atlanta (N.S. Shah);; University of KwaZulu-Natal and National Health Laboratory Service, Durban, South Africa (P. Moodley, K. Mlisana);; Albert Einstein College of Medicine and Montefiore Medical Center, Bronx, New York, USA (J.C.M. Brust)

**Keywords:** tuberculosis, drug resistance, South Africa, Georgia, pyrazinamide, sequencing, tuberculosis and other mycobacteria, bacteria

## Abstract

Although pyrazinamide is commonly used for tuberculosis treatment, drug-susceptibility testing is not routinely available. We found polymorphisms in the *pncA* gene for 70% of multidrug-resistant and 96% of extensively drug-resistant *Mycobacterium tuberculosis* isolates from South Africa and Georgia. Assessment of pyrazinamide susceptibility may be prudent before using it in regimens for drug-resistant tuberculosis.

Drug-resistant tuberculosis (TB) poses a significant threat to global health, with an estimated 480,000 new cases of multidrug-resistant tuberculosis (MDR TB) in 2014; 10% of these cases were classified as extensively drug-resistant tuberculosis (XDR TB) (*1*). MDR and XDR TB are associated with high mortality rates because of limited treatment options (*2**,**3*). Drug-susceptibility testing (DST) is critical for constructing MDR and XDR TB treatment regimens.

Pyrazinamide is a critical component of first-line TB regimens but is also recommended for use in drug-resistant TB regimens (*4*). Despite widespread use, phenotypic DST for pyrazinamide is not routinely performed because of the precise acidic conditions required (*5*). However, acidic environments also inhibit the growth of *Mycobacterium tuberculosis*, making phenotypic pyrazinamide DST challenging even in sophisticated TB laboratories. The need for pyrazinamide DST is underscored by the potential synergy between pyrazinamide and new TB drugs under study for treatment of drug-susceptible and drug-resistant TB (*6*).

The development of rapid molecular tests has simplified testing for drug resistance. Assays detect resistance-conferring mutations in genes associated with phenotypic resistance to isoniazid, rifampin, fluoroquinolones, ethambutol, aminoglycosides, and capreomycin. Genotypic testing for pyrazinamide may provide a simpler method for assessing drug susceptibility (*7*). Pyrazinamide resistance arises through genetic mutations in the *pncA* gene (*8*). *pncA* encodes pyrazinamidase, which converts pyrazinamide into pyrazinoic acid for its antimycobacterial activity. However, data on the frequency and diversity of *pncA* mutations in clinical settings are limited.

We characterized the frequency and diversity of polymorphisms in the *pncA* gene and estimated the prevalence of pyrazinamide resistance among patients with MDR and XDR TB in South Africa and the country of Georgia. Ethics approval for the study was obtained from Emory University, Albert Einstein College of Medicine, National Center for Tuberculosis and Lung Diseases, University of KwaZulu-Natal, and the Centers for Disease Control and Prevention.

## The Study

We performed a cross-sectional study examining *pncA* polymorphisms in *M. tuberculosis* isolates from a convenience sample of patients with MDR or XDR TB who were prospectively enrolled in studies from KwaZulu-Natal, South Africa (n = 451, diagnosed 2011–2014), and Georgia (n = 103, diagnosed November 2011–April 2012) (Technical Appendix). Cultures and DST were performed at the provincial TB reference laboratory in Durban, South Africa, and the National Reference Laboratory in Tbilisi, Georgia. Samples underwent PCR amplification followed by standard capillary sequencing of the *pncA* promoter and coding DNA sequence at the Public Health Research Institute in Newark, New Jersey, USA, as previously described (*9*). Polymorphisms were identified by alignment of nucleotide sequences to the H37Rv reference strain by using ClustalW2 (http://www.ebi.ac.uk/Tools/msa/clustalw2/). We calculated the frequency of each *pncA* polymorphism and classified mutations as synonymous or nonsynonymous. We compared polymorphisms with those reported in the literature to identify any that are known to be associated with phenotypic susceptibility and to determine the proportion that are likely to confer phenotypic resistance (*10**–**15*).

To determine the effect of clonal expansion of MDR and XDR TB on the diversity of *pncA* mutations, we also compared IS*6110*-based restriction fragment-length polymorphism (RFLP) patterns with *pncA* mutations for the isolates from South Africa (conducted at the Public Health Research Institute). The distribution of RFLP patterns among isolates with identical *pncA* mutations was examined to determine if the *pncA* mutation arose de novo or may have been transmitted.

We completed targeted *pncA* gene sequencing for 554 unique patient-isolates [1 isolate/patient], 167 MDR TB and 387 XDR TB; of these, 99 (59%) of MDR TB and 215 (56%) of XDR TB patients had previously received treatment for TB. A *pncA* polymorphism was found in 117 (70%) MDR TB and 370 (96%) XDR TB isolates (Tables 1, 2). The proportion of MDR TB and XDR TB isolates with *pncA* polymorphisms did not differ significantly between those from South Africa and Georgia (MDR, 68% vs. 72%, p = 0.74; XDR, 96% vs. 90%, p = 0.73, respectively).

**Table 1:** *pncA* gene sequencing and RFLP results of 74 MDR TB and 377 XDR TB isolates from KwaZulu-Natal Province, South Africa*

Mutation	*pncA* mutation type†	Frequency (no. isolates)	RFLP‡
Mutations common to MDR TB and XDR TB isolates			
1	Ins C after 456	271 (MDR TB: 7; XDR TB: 264)	MDR TB: HP
			XDR TB: HP (263), mixed (1)
2	TTG(L) 151 TCG(S)§	19 (MDR TB: 8; XDR TB: 11)	MDR TB: GY
			XDR TB: GY
3	AGG(R) 154 GGG(G)	17 (MDR TB: 3; XDR TB: 14)	MDR TB: AH
			XDR TB: AH
4	GCG(A) 102 GTG(V)§	15 (MDR TB: 13; XDR TB: 2)	MDR TB: BW (1), KO (12)
			XDR TB: BM (1), KO (1)
5	CAT(H) 71 TAT(Y)§	13 (MDR TB: 3; XDR TB: 10)	MDR TB: BH (1), BW (2)
			XDR TB: BH (1), BW (8), HP (1)
6	Ins A after 408§	8 (MDR TB: 1; XDR TB: 7)	MDR TB: CC
			XDR TB: CC (6), GY (1)
7	TCC(S) 59 CCC(P)§	7 (MDR TB: 4; XDR TB: 3)	MDR TB: BF
			XDR TB: BF
8	GGT(G) 132 GCT(A)§	4 (MDR TB: 1; XDR TB: 3)	MDR TB: HP
			XDR TB: HP
9	GGT(G) 97 GAT(D)§	2 (MDR TB: 1; XDR TB: 1)	MDR TB: KM
			XDR TB: M
Mutations specific to MDR TB isolates			
10	CTG(L) 35 CTA(L)¶	1	W
11	CTG(L) 35 CTA(L)¶	1	GY
	TTG(L) 151 TCG(S)		
12	TTC(F) 58 TCC(S)§	1	BH
13	ACT(T) 76 ATT(I)§	1	CC
14	GAG(E) 91 CAG(Q)	1	BH
15	GGT(G) 97 TGT(C)§	1	H
16	Ins A after 407§	1	CC
17	Del CAGGGTGC at 459	1	W
18	Del T at 515§	1	GO
Mutations specific to XDR TB isolates			
19	insG after 515	23	MH
20	TAC(Y) 34 GAC(D)§	5	BH
21	GTG(V) 139 GGG(G)§	3	HP
22	GTG(V) 130 GCG(A)#	2	W
23	Del G at 385, TTG(L) 151 TCG(S)	2	GY
24	GAG(E) 15 GGG(G)	1	W
25	ACC(T) 47 ATC(I)	1	KR
26	CAC(H) 51 CCC(P) §	1	HP
27	TCC(S) 65 TCT(S)¶,#	1	GD
28	TGC(C) 104 CGC(R)	1	MH
29	ACC(T) 153 CAC(H)§	1	HP
30	AGG(R) 154 TGG(W)§	1	HP
31	Ins G after 312	1	HP
32	Ins G after 313	1	HP
33	Del T at 389	1	HP
34	Ins C after 456, CTG(L) 35 CTA(L)¶	1	HP
Wild-type		40 (MDR TB: 24; XDR TB: 16)	MDR TB: BE (2), BH (4), BM (1), BW (1), CC (2), FO (1), GO (1), GY (1), HZ (1), MH (2), W (8); XDR TB: AH (1), BM (1), BW (1), CC (1), GY (1), HP (2), MH (4), mixed (1), W (4)

*del, deletion; ins, insertion; MDR, multidrug resistant; RFLP, restriction fragment-length polymorphism; SNP, single-nucleotide polymorphism; TB, tuberculosis; XDR, extensively drug-resistant. Row colors: pink, frameshift mutations (insertions or deletions); green, synonymous mutations or SNPs reported as being associated with phenotypic susceptibility to pyrazinamide; gray, SNPs reported as being associated with phenotypic resistance to pyrazinamide; blue, single-nucleotide polymorphisms not previously reported in the literature. †Insertions and deletions are presented with the nucleotide position where the polymorphism occurred; SNPs are presented with the codon position and the original and mutated 3 nucleotides and amino acid. ‡The nomenclature used for classifying IS6110 RFLP patterns was as follows: 2 isolates with an identical *IS*6110 banding pattern were assigned the same arbitrary 1- or 2-letter code (e.g., W, HP, or AB), which started with the first observed cluster, strain A (several decades ago). *IS*6110 patterns that were similar but not identical were denoted by the addition of a number (e.g., BE1, W4, or HP81). This table shows only the letter designations; numbers have been omitted for simplicity. Strains within each of the 2-letter designations had similar RFLP patterns.  §Reported in the literature as being associated with resistance to pyrazinamide. ¶Synonymous mutation. #Reported in the literature as being associated with susceptibility to pyrazinamide.

**Table 2:** *pncA* gene sequencing results of 93 MDR TB and 10 XDR TB isolates from Georgia*

Mutation	*pncA* mutation type†	Frequency (no. isolates)
Mutations specific to MDR TB isolates		
1	CAG(Q) 141 CCG(P)‡	20
2	CAT(H) 71 CGT(R)‡	8
3	CAT(H) 71 CCT(P)‡	6
4	Ins C after 420, 421ΔA, CAG(Q) 141 CCG(P)‡	3
5	TGG(W) 119 TTG(L)‡	3
6	ATC(I) 6 CTC(L)§	2
7	GAC(D) 49 GGC(G)‡	2
8	CCA(P) 69 CGA(R)‡	2
9	GTG(V) 155 GCG(A)§	2
10	TTG(L) 4 TGG(W)‡	1
11	ATC(I) 5 ACC(T)§	1
12	CTG(L) 27 CCG(P)‡	1
13	GAC(D) 49 GAG(E)‡	1
14	CAC(H) 51 TAC(Y)‡	1
15	CCG(P) 54 CAG(Q)‡	1
16	GAC(D) 63 GCC(A)‡	1
17	TCG(S) 66 CCG(P)‡	1
18	CAT(H) 71 CCT(P); Ins G after 547 (after G in GTT)	1
19	GGT(G) 97 AGT(S)‡	1
20	TAC(Y) 103 TAA(Ter)‡	1
21	TAC(Y) 103 TAG(Ter)‡	1
22	GCC(A) 134 CCC(P)	1
23	ACG(T) 142 ATG(M)‡	1
24	GGT(G) 162 TGT(C)	1
25	TGA(Ter) 187 CGA(R)§	1
26	Del A at 298	1
27	Ins A after 389 (after T in GTG)	1
28	Ins G after 449 (after sec G in GGC)	1
Wild type		26
Mutations specific to XDR TB isolates		
1	GTC(V) 7 GCC(A)‡	2
2	ACC(T) 47 AGC(S)‡	2
3	Ins TCT after 40 (after T In TGC)	1
4	GGC(G) 78 GAC(D)‡	1
5	GGT(G) 97 CGT(R)‡	1
6	TAC(Y) 103 GAC(D)‡	1
7	GGC(G) 105 CGC(R)	1
Wild type		1

*del, deletion; ins, insertion; MDR, multidrug resistant; SNP, single-nucleotide polymorphism; TB, tuberculosis; XDR, extensively drug resistant. Row colors: pink, frameshift mutations: insertions or deletions; green, synonymous mutations or SNPs reported as being associated with phenotypic susceptibility to pyrazinamide; gray, SNPs reported as being associated with phenotypic resistance to pyrazinamide; blue, SNPs not previously reported in the literature. †Insertion and deletions are presented with the nucleotide position where the polymorphism occurred; single nucleotide polymorphisms are presented with the codon position and the original and mutated three nucleotides and amino acid ‡Reported in the literature as being associated with resistance to pyrazinamide. §Reported in the literature as being associated with susceptibility to pyrazinamide.

A total of 69 distinct *pncA* polymorphisms were identified (Tables 1 and 2). Of these, 12 were insertions (313 patient-isolates), 5 were deletions (6 patient-isolates), and 52 were single-nucleotide polymorphisms (SNPs; 168 patient-isolates); all but 2 of these were nonsynonymous. No polymorphism was found in common between the isolates from South Africa and Georgia. Among the *pncA* SNPs identified, only 6 (9 patient-isolates) have previously been associated with phenotypic pyrazinamide susceptibility (*10**–**12*); 40 SNPs have been associated with phenotypic pyrazinamide resistance (146 patient-isolates), and 7 SNPs were not previously reported (23 patient-isolates) (*10**–**15*).

There were 34 polymorphisms identified from South Africa, of which 14 (41%, constituting 388 patient-isolates) were present in >1 patient (Table 1). We found that, for 382 (98%) of 388 patients, the RFLP pattern was identical to that of at least 1 other patient with the same *pncA* mutation (Table 1). Moreover, each *pncA* polymorphism was associated with only 1 RFLP pattern in 10 of the 14 polymorphisms. By comparison, 13 RFLP patterns were seen among the 40 patients with a wild-type *pncA* sequence.

## Conclusions

In this study, we found that 70% of MDR TB and 96% XDR TB patient-isolates had *pncA* polymorphisms. Given the high likelihood of frameshift mutations resulting in resistance and the high specificity (94%–98%) of *pncA* SNPs for pyrazinamide resistance (*13**,**14*), we estimate that at least 56%–66% of MDR TB and 90%–95% of XDR TB cases from these settings are likely to be resistant to pyrazinamide. Only a small number of mutations were synonymous, previously associated with pyrazinamide susceptibility, or had an SNP for which phenotypic susceptibility has not been previously tested. This finding has implications regarding the effectiveness of empiric use of pyrazinamide for drug-resistant TB or novel treatment regimens. Further studies are needed to fully determine the association of *pncA* mutations with treatment outcomes.

A diversity of *pncA* mutations—69 distinct polymorphisms—were observed among MDR and XDR TB patients, of which none were shared in common between the isolates from South Africa and Georgia. Most *pncA* polymorphisms were unique to individual patients. When the same *pncA* polymorphism was seen in >1 patient, the IS*6110* RFLP pattern was nearly always similar, suggesting that the *pncA* mutation was acquired before transmission. The diversity of polymorphisms underscores previous findings that there is no clear hotspot for *pncA* mutations (*12*), unlike resistance-conferring regions for other TB drugs (e.g., *rpoB*, *katG*) (*11*). Development of rapid molecular tests for pyrazinamide susceptibility may be hampered by the lack of a hotspot for mutations; 1 assay has been developed to detect the full wildtype *pncA* sequence, but its diagnostic accuracy has not yet been adequately tested (*7*).

A limitation of our study is that phenotypic pyrazinamide susceptibility testing was not performed on the sequenced isolates. Nonetheless, correlation with phenotypic testing has been previously reported in the literature for most polymorphisms, enabling us to estimate the proportion likely to be pyrazinamide resistant (*10**–**15*). In addition, the studies that provided these isolates were not specifically designed to be representative of all diagnosed cases of drug-resistant TB; nonetheless, the study populations were carefully selected to provide a high level of generalizability to the broader population. National drug resistance surveys that include pyrazinamide genotypic and phenotypic susceptibility should be designed to confirm these findings.

The high prevalence of *pncA* polymorphisms from geographically disparate countries suggests that guidelines to empirically use pyrazinamide in drug-resistant TB regimens, including shorter MDR TB regimens (*4*), should be reconsidered. Simplified assays to test pyrazinamide susceptibility are needed, although they may be difficult to develop given the genotypic or phenotypic complexities. Considering the potential synergy of pyrazinamide with new TB drugs, routine assessment of pyrazinamide will be increasingly necessary and useful.

Technical Appendix Supplemental methods for study of *pncA* gene mutations associated with pyrazinamide resistance in drug-resistant tuberculosis, South Africa and Georgia.
