# Supplementary material for: pncA Gene Mutations Associated with Pyrazinamide Resistance in Drug-Resistant Tuberculosis, South Africa and Georgia
Source: Emerg Infect Dis. 2017 Mar;23(3):491–5. doi: 10.3201/eid2303.161034 (PMC5382742; doi:10.3201/eid2303.161034)
Supplement: Technical Appendix — Supplemental methods for study of pncA gene mutations associated with pyrazinamide resistance in drug-resistant tuberculosis, South Africa and Georgia. [file 16-1034-Techapp-s1.pdf]

Article DOI: http://dx.doi.org/10.3201/eid2303.161034

pncA Gene Mutations Associated with Pyrazinamide Resistance in Drug-Resistant Tuberculosis, South Africa and Georgia

Technical Appendix

Supplemental Methods

Setting

In KwaZulu-Natal province, South Africa, the MDR TB and XDR TB incidences are 64 cases and 7.3 cases/100,000 population, respectively, and more than 80% of TB cases are HIV co-infected.(*1*) In Georgia, the proportion of new and retreatment TB cases that are MDR TB is 12% and 39% respectively, and 10% of MDR TB cases are XDR TB.(*2*) Fewer than 5% of TB cases are HIV co-infected. At the time of the study in both South Africa and Georgia, the standardized MDR and XDR TB regimens included PZA, but PZA DST was not routinely available in either setting.

Study Population from Parent Studies

Sputum samples were obtained from a convenience sample of newly diagnosed and retreatment MDR- and XDR TB patients prospectively enrolled in observational studies: SHOUT MDR TB and TRAX XDR TB studies in KwaZulu-Natal province; and a study examining the performance of the MTBDRsl assay in the Country of Georgia.(*3*–*5*) XDR TB participants in the TRAX study (n = 404) were enrolled from throughout KwaZulu-Natal province (*4*). Enrolled participants were geographically representative of the 1029 XDR TB patients diagnosed in the province during the study period (May 2011 – August 2014). MDR TB participants in the SHOUT MDR TB study (n = 206) were enrolled from three of the seven MDR TB treatment centers present in KwaZulu-Natal province at that time (*3*). The centers represented rural, semi-urban and urban districts in the province (Greytown, Port Shepstone, and Durban, respectively). A convenience sample of 206 participants was enrolled at these sites from among the estimated 6325 MDR TB patients who received treatment in the province during the study

period (September 2011 – November 2013. MDR TB and XDR TB patients (n = 159) were consecutively enrolled in Georgia from the National TB Reference Laboratory (NRL) and were representative of the 165 isoniazid- and rifampin-resistant TB patients diagnosed in all of Georgia (except West Georgia) during the study period (November 2011 – April 2012).(*5*)

Laboratory Methods

Sputum samples were cultured and had DST performed using Mycobacterial Growth Indicator Tube (MGIT) 960 broth culture (South Africa) or Lowenstein-Jensen agar (Georgia) in the parent studies. DST was performed for isoniazid, rifampin, streptomycin, kanamycin and ofloxacin at both sites at the KwaZulu-Natal provincial TB reference laboratory, National Health Laboratory Services (NHLS, South Africa) and National Reference Laboratory, National Center for Tuberculosis and Lung Disease (Tbilisi, Georgia); capreomycin DST was also conducted in Georgia.

Isolates from all participants in the parent study were eligible for the current study, and were successfully regrown for 74 MDR TB and 377 XDR TB participants from South Africa and 93 MDR TB and 10 XDR TB participants from Georgia. DNA was extracted and was subject to PCR amplification followed by standard capillary sequencing of the *pncA* promoter and coding DNA sequence at the Public Health Research Institute/Rutgers in New Jersey, as previously described.(*6*) Polymorphisms were identified by alignment of nucleotide sequences to the H37Rv reference strain (NCBI accession number AL123456) using ClustalW2.

References

1. Ndjeka N. Multi-Drug Resistant Tuberculosis: Strategic Overview on MDR TB Care in South Africa. 2014 [cited 2016 5/17/2016]; Available from: https://www.health-e.org.za/wp-content/uploads/2014/03/Strategic_overview_of_MDR_TB_RSA.pdf

2. World Health Organization. Global Tuberculosis Report 2015. Geneva. ; 2015. Report No.: WHO/HTM/TB/2015.22.

3. Brust JCM, Shah NS, Allana S, Mthiyane T, Mlisana K, Moodley P, et al. High survival and treatment success with concurrent MDR TB and HIV treatment in KwaZulu-Natal, South Africa. 46th Union World Conference on Lung Health. Cape Town: Int J TB Lung Dis; 2015.

4. Shah NS, Auld SC, Brust JCM, Mathema B, Ismail NA, Moodley P, et al. Transmission of Extensively Drug-resistant Tuberculosis in South Africa N Engl J Med. 2016;(provisionally accepted).

5. Tukvadze N, Bablishvili N, Apsindzelashvili R, Blumberg HM, Kempker RR. Performance of the MTBDRsl assay in Georgia. Int J Tuberc Lung Dis. 2014;18:233–9. PubMed http://dx.doi.org/10.5588/ijtld.13.0468

6. Sreevatsan S, Pan X, Stockbauer KE, Connell ND, Kreiswirth BN, Whittam TS, et al. Restricted structural gene polymorphism in the Mycobacterium tuberculosis complex indicates evolutionarily recent global dissemination. Proc Natl Acad Sci U S A. 1997;94:9869–74. PubMed http://dx.doi.org/10.1073/pnas.94.18.9869
